# Serology survey against multiple SARS-CoV-2 variants of residents in Tainan, Taiwan

**DOI:** 10.1080/21505594.2026.2659420

**Published:** 2026-04-14

**Authors:** I-Lin Hsu, Pin-Xian Du, Ting-Mao Chou, Chung-Che Cheng, Pei-Shan Tsai, Hsi-Chang Shih, Shu-Chuan Chen, Chung-Yi Li, Tzong-Shiann Ho, Guan-Da Syu

**Affiliations:** a Cross-College Elite Program, National Cheng Kung University, Tainan, Taiwan; b Department of Biotechnology and Bioindustry Sciences, National Cheng Kung University, Tainan, Taiwan; c Department of Surgery, National Cheng Kung University Hospital, Tainan, Taiwan; d Department of Pharmacology and Molecular Sciences, Johns Hopkins University School of Medicine, Baltimore, MD, USA; e Public Health Bureau, Tainan City Government, Tainan, Taiwan; f Department of Public Health, College of Medicine, National Cheng Kung University, Tainan, Taiwan; g Department of Public Health, College of Public Health, China Medical University, Taichung, Taiwan; h Department of Healthcare Administration, College of Medical and Health Science, Asia University, Taichung, Taiwan; i Department of Pediatrics, National Cheng Kung University Hospital, Tainan, Taiwan; j Department of Pediatrics, National Cheng Kung University Hospital Dou-Liou Branch, College of Medicine, National Cheng Kung University, Yunlin, Taiwan; k Center of Infectious Disease and Signaling Research, National Cheng Kung University, Tainan, Taiwan; l International Center for Wound Repair and Regeneration, National Cheng Kung University, Tainan, Taiwan; m Medical Device Innovation Center, National Cheng Kung University, Tainan, Taiwan

**Keywords:** Serology survey, COVID-19, SARS-CoV-2 variant, vaccine responses

## Abstract

**Background:**

The coronavirus disease 2019 (COVID-19) pandemic, caused by severe acute respiratory syndrome coronavirus 2 (SARS-CoV-2) and its evolving variants, continues to challenge global efforts to control its spread. Ongoing mutations may enhance transmissibility or lead to breakthrough infections, making immune monitoring crucial for public health policies.

**Methods:**

A SARS-CoV-2 variant protein microarray was used to assess total and neutralizing antibodies against multiple variants. A serological survey was conducted in October 2022 in Tainan, Taiwan, including general citizens and special medical care groups. The general population comprised residents from the city (n = 504), suburban (n = 166), and country areas (n = 88). Special groups included individuals in elderly care centers (n = 50), nursing homes (n = 51), kidney dialysis patients (n = 100), and people living with human immunodeficiency virus (PLWH) (n = 45). Statistical analyses were performed using one-way and repeated-measures analysis of variance (ANOVA).

**Results:**

The results demonstrated that the neutralization efficacy against Omicron and its subtypes declined according to the evolution of the variants, particularly in city and suburban areas. Special medical care populations, including individuals in elderly care centers, nursing homes, those undergoing kidney dialysis, and those PLWH, exhibited lower neutralization effectiveness. After analyzing the infection status, it can be found that special medical care populations had higher infection rates, especially in elderly care centers and nursing homes.

**Conclusions:**

SARS-CoV-2 protein microarray analysis is essential for assessing immunity at a population level. The findings highlight the need for adjusted vaccination strategies and ongoing surveillance of humoral immunity to address emerging SARS-CoV-2 variants effectively.

## Introduction

The coronavirus disease 2019 (COVID-19) pandemic is caused by the severe acute respiratory syndrome coronavirus 2 (SARS-CoV-2) [1], a beta coronavirus closely related to the human SARS-CoV virus [2–4]. The emergence of new variants of SARS-CoV-2 in the past few years has posed a significant challenge to the ongoing pandemic [5,6]. The global epidemiological trends of SARS-CoV-2 exhibit diversity, influenced by the response measures of different countries, the strength of healthcare systems, and the progress of vaccination. As of April 2024, SARS-CoV-2 has rapidly spread worldwide, resulting in over 704 million confirmed cases and 7 million deaths [7]. Various factors, including testing levels, population density, and public health measures, influence the confirmed cases and death figures of COVID-19 in each country [8].

Coronaviruses, like most RNA viruses, evolve rapidly. However, in the initial 8 months, the virus seemed to show limited apparent evolution. Through rapid transmission, variants of concern (VOC) such as Alpha, Beta, Gamma, Delta, Omicron, and their subtypes have emerged [9]. Starting from the Beta variant, the first known variants exhibit immune escape phenomena, rendering earlier vaccines less effective against newer variant strains [10–16]. Omicron and its subtypes have shown an increase in transmission and breakthrough infections, which poses a significant challenge to public health [17–20]. With effective epidemic control in Taiwan, the Omicron cases were first reported in January 2022, became substantial in April 2022, and were transmitted to 16% of the population in July 2022 (CDC, Taiwan). As of October 2022, 33% of COVID-19 cases were accumulated, and BA.5 (96%) was the dominant strain in Taiwan (CDC, Taiwan). Facing the ongoing challenge of the COVID-19 pandemic becoming endemic, the uncertainty surrounding the transmissibility and pathogenicity of new variants remains unknown, emphasizing the importance of testing and continuous monitoring [21].

Humoral immunity is the antibody-mediated immune response, mainly produced by B cells [22]. Specific antibodies circulating in the blood may help to fight against invading pathogens through neutralization, opsonization, and complement activation, which are opposite from cell-mediated immunity. Accordingly, analytical platforms capable of profiling antibody responses at large scale are required. Protein microarrays, highly sensitive and high-throughput detection tools, are suitable for large-scale screening due to their ability to immobilize various antigens and profile humoral responses [23]. We have developed different protein microarrays to analyze serum antibodies for COVID-19 patients, COVID-19 patients with different severities, healthy subjects with different COVID-19 vaccines, and patients with COVID-19 vaccines [24–26]. In October 2022, Taiwan was still under pandemic control measures and needed humoral immunity data for public health and policy-making. To measure the humoral immunity against multiple variants, we adapted the previously developed SARS-CoV-2 variant (CoVariant) protein microarrays to profile the neutralizing antibodies and binding antibodies against multiple SARS-CoV-2 variants, including Wild-type (WT), B.1.1.7 (Alpha), B.1.351 (Beta), P.1 (Gamma), B.1.617.2 (Delta), B.1.617.3, B.1.1.529 (Omicron), BA.2.12.1 (Omicron), BA.4 (Omicron), and BA.5 (Omicron) [24,26,27]. We investigated serum samples from 1,004 subjects in Tainan, Taiwan, including subjects from different population densities and subjects with special medical conditions. With CoVariant protein microarrays, we aim to report the neutralizing activities, binding antibodies, and infection rates against multiple SARS-CoV-2 variants in Tainan, Taiwan.

## Materials and methods

### Fabrication of CoVariant protein microarray and quality control

**Figure 1.**
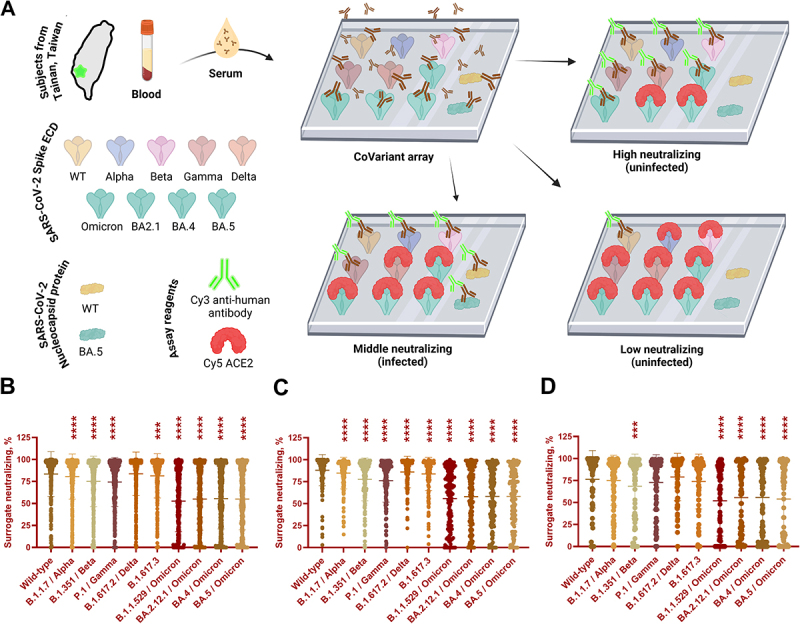
Neutralizing responses to SARS-CoV-2 and its variants from subjects in different Tainan areas.

The concept of the CoVariant Protein Microarrays and their assay procedures are shown in Figure 1(A). The procedures for fabricating CoVariant protein microarray and quality control were described in our previous publications [24,25]. To produce multiplexed CoVariant protein microarrays, the receptor binding domain (RBD) of WT and variant spike proteins, the extracellular domain (ECD) of WT and variant spike proteins, WT and omicron nucleocapsid proteins, positive controls, and negative controls were printed on aldehyde slides (Table S1). Each sample was printed in triplicate per block and 14 identical blocks per slide using a contact printer (CapitalBio, #SmartArrayer 136). The arrays were immobilized overnight and stored at −80°C.

(A) The CoVariant protein microarray contained multiple SARS-CoV-2 spike and nucleocapsid proteins for quantifying binding and surrogate neutralizing antibodies. The binding antibodies from serum were quantified by Cy3-labeled anti-human antibody. Antibodies against nucleocapsid proteins reflected the previous infections (lower middle panel). The surrogate neutralizing antibodies were quantified by inhibiting Cy5-labeled ACE2 binding to spike ECDs (right panels). Sera from subjects in different Tainan areas and medical conditions were collected and analyzed. Created in https://BioRender.com. (B) city (*n* = 504), (C) suburban (*n* = 166), and (D) country (*n* = 88). Data were analyzed by one-way ANOVA with repeated measures followed by Dunnett’s posttests, whereas ***p < 0.001 and ****p < 0.0001 compared to WT.

Assessment of protein immobilization and functional activity involved blocking the array with SuperBlock (Thermo; Lot# VG299792) for 15 min with shaking at 50 rpm. The array was then incubated with biotinylated Angiotensin-converting enzyme 2 (ACE2, Sino Biological, #10108-H08H-B) in a serial dilution starting at 0.125 ng/mL, diluted 2-fold, and incubated for 1 h. The array was then incubated with Cy5-labeled streptavidin (Jackson Lab, #016–170-084) at 1.8 µg/mL or Cy5-labeled anti-His (Jackson Lab, #300–605-240) at 1.1 µg/mL for an additional 1 h. Afterward, the array was rinsed five times, dried, and scanned using the SpinScan system (Caduceus Biotechnology) with PMT settings at Red 30% and Green 25%.

### Subjects

A seroepidemiological survey was implemented in Tainan City in October 2022 to assess the prevalence of community-level SARS-CoV-2 infection and to characterize humoral immune responses following COVID-19 vaccination, with the objective of guiding subsequent public health interventions. Informed consent was obtained from all adult participants, while parental or legal guardian consent, along with age-appropriate assent, was secured for minors capable of providing it, following a thorough explanation of the study’s objectives and procedures. The consent statement was signed in writing.

Participants also consented to the secondary use of de-identified data for further analysis. All collected data were securely stored within the Biological Database of the Tainan City Government Health Bureau. De-identified neutralizing antibody test results derived from this repository were utilized for the present analysis. The study was conducted in accordance with the ethical principles outlined in the Declaration of Helsinki and received approval from the Institutional Review Board of National Cheng Kung University Hospital (IRB No. B-ER-112–080). To comprehensively assess the immunity status against COVID-19 and its variants among residents of Tainan City, the study stratifies participants into two primary cohorts: special populations and the general population. The special populations encompass institutional residents, people living with human immunodeficiency virus (PLWH), and dialysis patients, comprising a total of 246 individuals. These groups were selected due to their distinct health conditions requiring specialized attention. The general population sample, consisting of 758 individuals, was stratified based on demographic proportions and statistical requirements, with specific distributions across various age groups and regional characteristics. This sampling methodology ensures representativeness, thereby enhancing the study’s credibility. Testing was concentrated in designated areas to streamline sample collection and mitigate logistical challenges.

### Serum profiling

Serums were collected and stored at −80°C until profiled. The CoVariant arrays were blocked via SuperBlock (Thermo; Lot# VG299792) for 15 min with shaking at 50 rpm. Following blocking, the CoVariant arrays were exposed to 50 μL of 50-fold diluted serum in 1% bovine serum albumin in tris buffered saline with tween 20 (TBST) for 1 hour, followed by washing 3 times in TBST for 10 min. Subsequently, arrays were incubated with 50 μL of biotinylated human ACE2 at a concentration of 78 ng/mL (Sino biological, #10108-H08H – B), Cy5-conjugated streptavidin at 2 ng/mL (Jackson Lab, #016–170-084), and Cy3-labeled anti-human IgG+IgA+IgM antibodies at 62.5 ng/mL (Jackson Lab, #109–165-064) for another hour. After a final round of washing 3 times in TBST for 10 min, the arrays were dried and scanned to detect Cy3 and Cy5 signals.

### Data analysis

The obtained signals were analyzed using GenePix Pro software with foreground minus background calculation. Surrogate neutralizing activity was determined based on the inhibition of ACE2 binding, calculated using the formula: 1 – (ACE2 with serum/ACE2 without serum) × 100%. To quantify total antibody (IgG+IgA+IgM), fluorescence signals from spike proteins were divided by their corresponding anti-His signals to normalize the protein amounts. Statistical comparisons between multiple groups were conducted using one-way ANOVA, followed by Tukey’s post-hoc tests. Statistical comparisons within a group for multiple variants were conducted using one-way ANOVA with repeated measures, followed by Dunnett’s posttests. A *p*-value less than 0.05 was considered statistically significant. Statistical analyses and figure generation were performed using GraphPad Prism software. All data were presented as mean ± standard deviation (SD), with “n” representing the number of subjects.

## Results

In October 2022, Tainan entered the community transmission of Omicron variants, led by BA.5 (96%). The new or accumulated COVID-19 cases increased dramatically (Figure S1A-B, yellow box). The cumulative number of confirmed cases was approximately half a million, accounting for 30% of the Tainan population (Figure S1A). We recruited subjects from different Tainan areas to survey the vaccine efficacies and infection rates. In the general population living in the different Tainan areas, we collected 758 sera from city residents (*n* = 504), suburban residents (*n* = 166), and country residents (*n* = 88) in proportion to the population. The baseline characteristics, including sample size, gender, age, and COVID-19 vaccine shots, were summarized in Table S2.

Since subjects with special medical conditions are more vulnerable to infection and with altered COVID-19 vaccine responses, we also recruited them to study the vaccine efficacies and infection rates. In subjects with special medical conditions, we collected 246 sera from the elderly care center (*n* = 50), nursing home (*n* = 51), dialysis (*n* = 100), and PLWH (*n* = 45) in Tainan. In Table S2, we summarized the baseline characteristics of the subjects for the sample size, gender, age, and COVID-19 vaccine shots.

**Figure 2.**
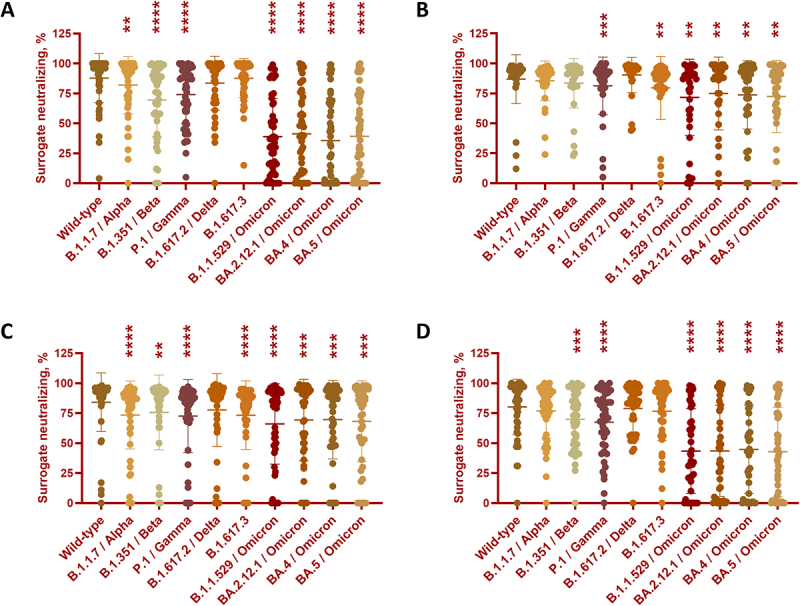
Neutralizing responses to SARS-CoV-2 and its variants from Tainan residences with different medical conditions.

The CoVariant protein microarray is a high throughput platform to interrogate vaccine responses and previous infections, and we updated the CoVariant arrays to BA.5 (the major variant during the survey) and applied them in this serology survey (Figure 1(A)). There were three major sets of data generated by the CoVariant protein microarray, e.g. surrogate neutralizing against spike from multiple variants, binding antibody against spike from multiple variants, and binding antibody against nucleocapsid from multiple variants (summarized in Tables S3 and S4). In the general population, the surrogate neutralizing was generally lowered against Beta, Omicron, and Omicron subvariants (Figure 1(B–D)). The surrogate neutralizing against WT ECD was higher in the city (83.5 ± 25.4%) and suburban (88.1 ± 17.7%) than in the country (76.4 ± 32.5%) (Figure S2A). We further analyzed the dominant BA.5 and showed similar surrogate neutralizing in the city (55 ± 34.9%), the suburban (58.2 ± 31.3%), and the country (53.9 ± 41.2%) (Figure S3A). In subjects with special medical conditions, the surrogate neutralizing was generally lowered against Gamma, Omicron, and Omicron subvariants (Figure 2). The surrogate neutralizing against WT ECD was similar in the elderly care center (84.6 ± 23.4%), in the nursing home (84.1 ± 24.1%), in the dialysis (89.3 ± 19.1%), and in the PLWH (80.2 ± 22.8%) (Figure S2B). The surrogate neutralizing against BA.5 ECD was higher in the elderly care center (72.7 ± 29.3%) and the nursing home (68.1 ± 33.4%) than the dialysis (51.4 ± 34.1%) and the PLWH (42.8 ± 36.9%) (Figure S3B).

Sera from Tainan residences with different medical conditions were collected and analyzed for their surrogate-neutralizing activities. (A) elderly care center (*n* = 50), (B) nursing home (*n* = 51), (C) dialysis (*n* = 100), and (D) PLWH (*n* = 45). Data were analyzed by one-way ANOVA with repeated measures followed by Dunnett’s posttests, whereas **p < 0.01, ***p < 0.001, and ****p < 0.0001 compared to WT.

**Figure 3.**
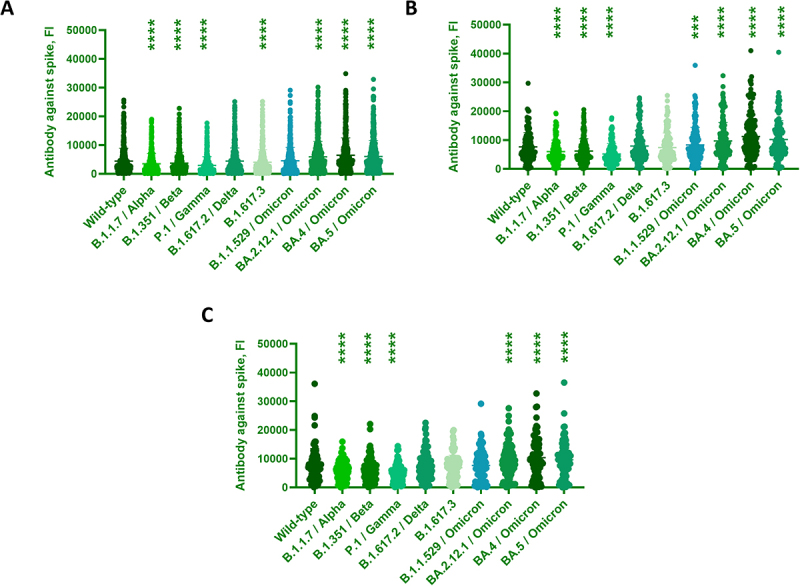
Antibody against SARS-CoV-2 and its variants from subjects in different Tainan areas.

**Figure 4.**
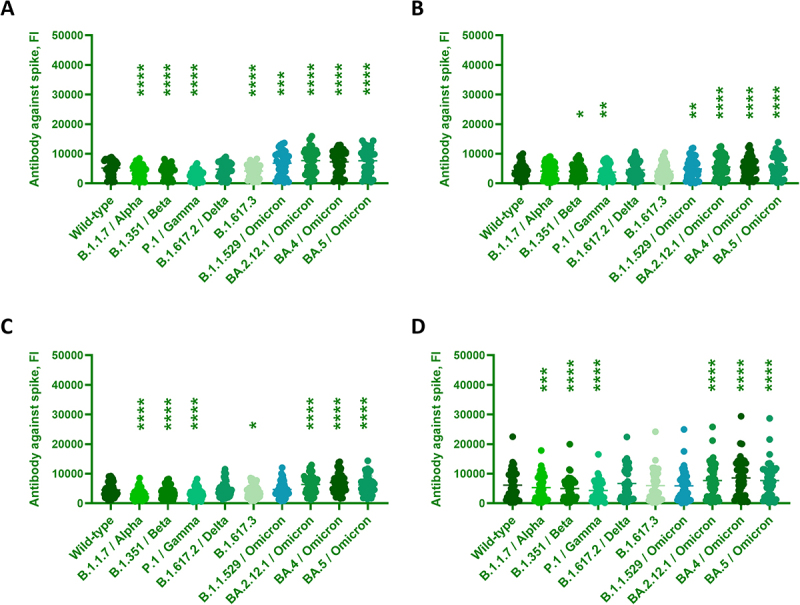
Antibody against SARS-CoV-2 and its variants from Tainan residences with different medical conditions.

This study also quantified the binding antibody against multiple spike variants to evaluate the seroconversion efficacies after vaccinations or infections. Compared to the WT, the overall binding antibody was lower against Alpha, Beta, and Gamma in the general population. Interestingly, the binding antibody was elevated against Omicron or its subvariants compared to the WT ECD (Figure 3). The binding antibody against WT ECD was increased in the suburban (7628.9 ± 4961.6 FI) and country (7693 ± 5832.1 FI) than in the city (4431.3 ± 4480.2 FI) (Figure S2C). Similarly, the binding antibody against the dominant BA.5 was elevated in the suburban (10187 ± 6540.2 FI) and country (9650.1 ± 6300.9 FI) than in the city (6049.7 ± 5582.9 FI) (Figure S3C). Compared to the WT, subjects with special medical conditions inhibited the overall binding antibody against Beta and Gamma but enhanced against Omicron and its subvariants (Figure 4). The binding antibody against WT ECD was higher in the PLWH (6156.3 ± 4759.5 FI) than in the dialysis (3333 ± 2133.5 FI), nursing home (3820.2 ± 2963.1 FI), and elderly care center (4320.3 ± 2496.2 FI) (Figure S2D). Similarly, the binding antibody against the dominant BA.5 was evoked in the PLWH (7700.2 ± 5789.2 FI) than in the dialysis (4719.7 ± 2872 FI) (Figure S3D).

Sera from subjects in different Tainan areas were collected and analyzed for their total antibody (IgG+IgA+IgM) bindings. (A) city (*n* = 504), (B) suburban (*n* = 166), and (C) country (*n* = 88). Data were analyzed by one-way ANOVA with repeated measures followed by Dunnett’s posttests, whereas ***p < 0.001 and ****p < 0.0001 compared to WT.

Sera from Tainan residences with different medical conditions were collected and analyzed for their total antibody (IgG+IgA+IgM) bindings. (A) elderly care center (*n* = 50), (B) nursing home (*n* = 51), (C) dialysis (*n* = 100), and (D) PLWH infection (*n* = 45). Data were analyzed by one-way ANOVA with repeated measures followed by Dunnett’s posttests, whereas *p < 0.05, **p < 0.01, ***p < 0.001, and ****p < 0.0001 compared to WT.

**Figure 5.**
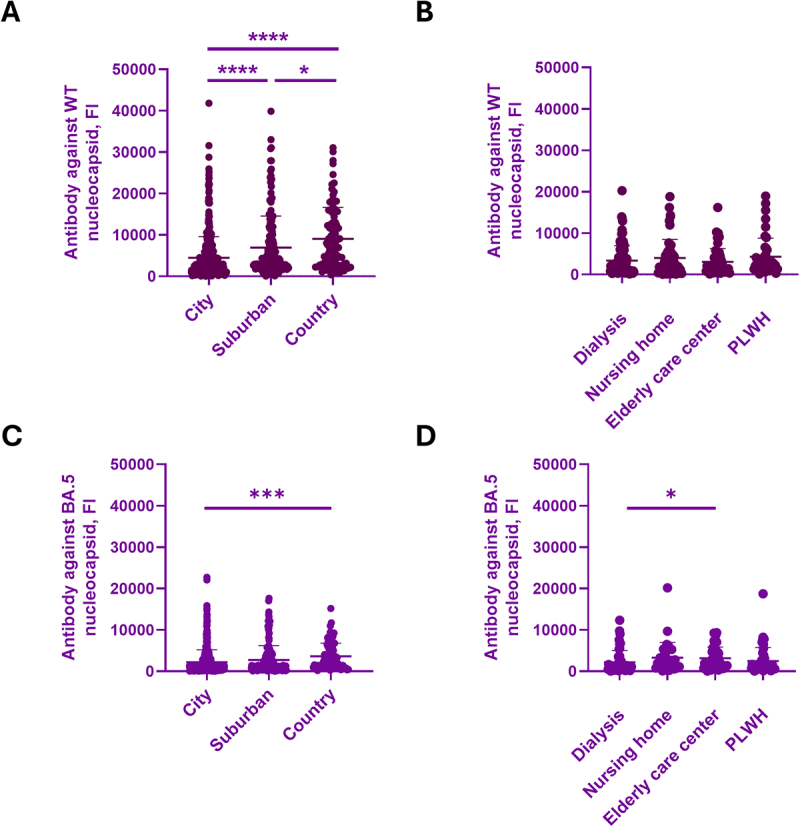
Antibody against wt or Omicron nucleocapsid (infection) in general and special medical care groups.

Current vaccines are designed against the spike protein, so analyzing the presence of antibodies against the nucleocapsid protein in the blood can indicate whether the subjects were previously infected. In the general population, the antibody against WT nucleocapsid was highest in country areas (9028.6 ± 7560.9 FI), followed by suburban areas (6926 ± 7599.7 FI), and the lowest in city areas (4455.2 ± 5127.5 FI) (Figure 5(A)). The antibody against omicron nucleocapsid was elevated in the country (3618.7 ± 3146.1 FI) than in city areas (2198.5 ± 3003.3 FI) (Figure 5(C)). In the special medical care population, the antibody against WT nucleocapsid was similar in dialysis (3353.6 ± 3584.6 FI), nursing homes (3995 ± 4439 FI), elderly care centers (3085.7 ± 3186.5 FI), and PLWH (4282.3 ± 4440.7 FI) (Figure 5(B)). The antibody against omicron nucleocapsid was elevated in dialysis (3152.1 ± 2670.2 FI) than in elderly care centers (1848.7 ± 2226.3 FI) (Figure 5(D)).

Sera from (A, C) the general population in different Tainan areas and (B, D) the special medical care groups were analyzed for their serum antibody against WT or Omicron nucleocapsid proteins. Data were analyzed by one-way ANOVA followed by Tukey’s posttests. *p < 0.05 and ****p < 0.0001.

## Discussion

Expanding discussions on infection investigations and vaccine administration for emerging infectious diseases requires a multifaceted approach to address the complexity associated with these health threats. At that time, epidemic control measures were still necessary. Besides understanding transmission dynamics, comprehensive research is crucial for developing effective vaccine and treatment strategies. In vaccine administration, staying ahead of rapidly evolving pathogens demands continuous research and development efforts. This includes identifying potential antigens, testing candidate vaccines, and ensuring their safety and efficacy. Furthermore, establishing robust vaccination programs is crucial for achieving widespread immunity and preventing large-scale outbreaks. For the adequacy of the vaccination program, large-scale screening is needed. Our previously developed CoVariant array is the optimal monitoring tool for this purpose. Our team has previously applied the CoVariant array to study COVID-19 severities, vaccine episodes, Kawasaki diseases, rheumatoid arthritis patients, and kidney transplantation recipients [24–26]. By incorporating extensive random sampling of prevalent variants and testing their antibody responses and neutralizing capabilities against them, we can gain insights into the regional antibody levels and infection status. This information allows for adjustments and improvements to the vaccination strategy.

Before the Omicron variant, most individuals exhibited antibody responses and neutralizing efficacy more significantly than 70% against the preceding variants. However, there has been an increase in antibody escape for variants emerging after Omicron [28,29]. There is a clear trend of decreasing neutralization effectiveness. However, based on a study in the United Kingdom, it is observed that Spikevax, targeting the BA.1 variant, can enhance neutralization against some variants emerging after Omicron [30]. Yet, evaluating the performance against BA.4/5 with the reinforced immune booster indicates a further immune escape compared to BA.1 [30]. Despite the increased immune escape for particular variants, vaccination with the updated vaccines can still help reduce the risk of infection. Therefore, encouraging the public to receive the new vaccines is advisable.

Investigating differences in infection rates across different regions can provide insights into healthcare measures. Variations in vaccination coverage, healthcare access, and environmental factors contribute significantly to these disparities, influencing the spread and severity of infections [31]. Understanding these regional differences can inform public health strategies and resource allocation [32]. In our study, lower infection rates were observed in subjects from Tainan city compared to the country areas. There are two potential reasons: population per household and medical accessibility. Due to pandemic control, the Taiwan CDC advises COVID-19 patients to stay home for quarantine, which may lead to in-house infections. According to the population data from the Bureau of Civil Affairs Tainan City Government, the population per household in city, suburban, and country is 2.41, 2.39, and 2.44 people, respectively [33]. Based on the population per household, it may explain higher infection rates in country areas. There is research on infectious disease diagnoses and how geographical differences influence city areas, with their advanced healthcare infrastructure and access to diagnostic tools, facilitating timely detection and response and reducing the spread of infections [34]. Country areas, where healthcare facilities are less accessible, often experience diagnostic delays, increasing infection risks similar to our research.

Population groups requiring special medical care typically have more comprehensive access to healthcare resources. The infection rates in elderly care centers and nursing homes are higher compared to dialysis and PLWH groups. This may be attributed to the communal living arrangements in elderly care centers and nursing homes, where individuals reside together, and the generally older age of the residents, leading to lower immunity and increased susceptibility to infections within the facility. Similar results are reported in other study [35]. Compared to Dialysis and PLWH groups, where individuals have more freedom of movement. Therefore, the closed-space contact is one of the significant risks of COVID-19 infection.

## Conclusion

This study comprehensively assesses SARS-CoV-2 immunity across diverse populations, including general citizens and vulnerable groups. Using a high-throughput protein microarray, it efficiently profiles neutralizing and binding antibodies against multiple variants, offering insights into vaccine effectiveness and breakthrough infections. The findings highlight regional differences in immunity and emphasize the need for targeted vaccination strategies. The adaptable microarray platform also enables ongoing surveillance of emerging variants, making it a valuable tool for public health monitoring. However, there is a lack of infection data and SARS-CoV-2 sequencing data, limiting our ability to correlate humoral responses to specific infection timing or strains.

## Supplementary Material

Supplementary file 2.xlsx

Supplementary file 1.docx

## Data Availability

The data supporting the findings of this study, including raw data and all supplementary materials, are openly available in the Figshare repository at https://doi.org/10.6084/m9.figshare.27174600
