# Supplementary material for: Serology survey against multiple SARS-CoV-2 variants of residents in Tainan, Taiwan
Source: Virulence. 2026 Apr 14;17(1):2659420. doi: 10.1080/21505594.2026.2659420 (PMC13097773; doi:10.1080/21505594.2026.2659420)
Supplement: Supplementary file 1.docx [file KVIR_A_2659420_SM3654.docx]

**Supporting Information**

**Table S1. Samples for fabricating CoVariant protein microarrays**

| No | Sample name | Catalog number | Provider |
| --- | --- | --- | --- |
| 1 | SARS-CoV-2 ECD | 40589-V08B1 | Sino biology |
| 2 | B.1.1.7 ECD | 40589-V08B5 | Sino biology |
| 3 | B.1.351 ECD | 40589-V08B9 | Sino biology |
| 4 | P.1 ECD | 40589-V08B8 | Sino biology |
| 5 | B.1.617.2 ECD | 40589-V08B16 | Sino biology |
| 6 | B.1.617.3 ECD | 40589-V08B17 | Sino biology |
| 7 | B.1.529 ECD | 40589-V08H26 | Sino biology |
| 8 | BA.2.12.1 ECD | SPN-C522d | Acro biosystem |
| 9 | BA.4 ECD | SPN-C5229 | Acro biosystem |
| 10 | BA.5 ECD | SPN-C522e | Acro biosystem |
| 11 | SARS-CoV-2 RBD | 40589-V08B33 | Sino biology |
| 12 | B.1.1.7 RBD | 40592-V08H82 | Sino biology |
| 13 | B.1.351 RBD | 40592-V08H85 | Sino biology |
| 14 | P.1 RBD | 40592-V08H86 | Sino biology |
| 15 | B.1.617.2 RBD | 40592-V08H90 | Sino biology |
| 16 | B.1.617.3 RBD | 40592-V08H115 | Sino biology |
| 17 | B.1.529 RBD | 40592-V08H121 | Sino biology |
| 18 | BA.2.12.1 RBD | 40592-V08H132 | Sino biology |
| 19 | BA.4 RBD | 40592-V08H130 | Sino biology |
| 20 | BA.5 RBD | 40592-V08-131 | Sino biology |
| 21 | SARS-CoV-2 N | 40588-V08B | Sino biology |
| 22 | B.1.529 N | 40588-V07E34 | Sino biology |
| 23 | Cy3 + Cy5 landmark | 109-005-064/ 109-165-148 | Jackson Laboratory |
| 24 | BSA | A7906 | Sigma |
| 25 | Protein A | P2165 | Sigma |

**Table S2. Baseline characteristics of general population and special medical care groups**

|  | City (n=504) | Suburban (n=166) | Country (n=88) | Elderly care center (n=50) | Nursing home (n=51) | Dialysis (n=100) | PLWH (n=45) |
| --- | --- | --- | --- | --- | --- | --- | --- |
| Gender Male (n, %) | 167 (33.1%) | 62 (37.3%) | 34 (38.6%) | 35 (70%) | 21 (41.2%) | 57 (57%) | 45 (100%) |
| Age (mean ± SD, years) | 41 ± 18.9 | 42.4 ± 20.4 | 36.9 ± 22.2 | 67.6 ± 15.8 | 72.1 ± 15.6 | 66 ± 11.3 | 38.6 ± 10 |
| Vaccine shots (mean ± SD, doses) | 3.3 ± 1 | 3.4 ± 0.9 | 2.8 ± 0.3 | 3.9 ± 0.3 | 2.9 ± 1.3 | 3.7 ± 1.5 | 3.5 ± 0.8 |
| At least 1 vaccine shot (n, %) | 494 (98%) | 164 (98.8%) | 81 (92%) | 50 (100%) | 45 (88.2%) | 92 (92%) | 45 (100%) |
| At least 2 vaccine shots (n, %) | 478 (94.8%) | 163 (98.2%) | 77 (87.5%) | 50 (100%) | 44 (86.3%) | 86 (86%) | 45 (100%) |
| At least 3 vaccine shots (n, %) | 424 (84.1%) | 142 (85.5%) | 57 (64.8%) | 50 (100%) | 40 (78.4%) | 84 (84%) | 43 (95.6%) |
| At least 4 vaccine shots (n, %) | 229 (45.4%) | 75 (45.2%) | 22 (25%) | 45 (90%) | 16 (31.4%) | 73 (73%) | 19 (42.2%) |
| At least 5 vaccine shots (n, %) | 30 (5.9%) | 17 (10.2%) | 5 (5.7%) | 1 (2%) | 4 (7.8%) | 35 (35%) | 6 (13.3%) |
| Unvaccinated (n, %) | 9 (1.8%) | 0 (0%) | 7 (8%) | 0 (0%) | 5 (9.8%) | 8 (8%) | 0 (0%) |
| *Vaccine data unavailable (n, %) | 1 (0.2%) | 2 (1.2%) | 0 (0%) | 0 (0%) | 1 (0.2%) | 0 (0%) | 0 (0%) |

*The 4 subjects without vaccination data might be due to ID recording or healthcare database errors

**Table S3. The surrogate neutralizing or binding antibody responses in different Tainan areas**

|  | City (n=504) | Suburban (n=166) | Country (n=88) |
| --- | --- | --- | --- |
| *CoVariant spike neutralizing antibody (nAb)* |  |  |  |
| SARS-CoV-2 ECD / WT nAb | 83.5 ± 25.4 | 88.1 ± 17.7 | 76.4 ± 32.5 |
| B.1.1.7 ECD / Alpha nAb | 80.5 ± 25.6 | 84.7 ± 17.9 | 75.1 ± 28.4 |
| B.1.351 ECD / Beta nAb | 75.1 ± 28.6 | 77.6 ± 24.2 | 68.5 ± 36.3 |
| P.1 ECD / Gamma nAb | 74.4 ± 27.7 | 76.2 ± 24.4 | 73 ± 31.2 |
| B.1.617.2 ECD / Delta nAb | 83.6 ± 24.7 | 86 ± 17.9 | 79 ± 26.7 |
| B.1.617.3 ECD / nAb | 81.4 ± 25.1 | 83.7 ± 18.7 | 73.8 ± 31 |
| B.1.1.529 ECD / Omicron nAb | 52.6 ± 34.8 | 55.9 ± 32.1 | 51.9 ± 39.8 |
| BA.2.12.1 ECD / Omicron nAb | 55 ± 35.3 | 57.9 ± 32.6 | 55.5 ± 38.5 |
| BA.4 ECD / Omicron nAb | 55.6 ± 34.4 | 58.3 ± 31.2 | 55.7 ± 39.4 |
| BA.5 ECD / Omicron nAb | 55 ± 34.9 | 58.2 ± 31.3 | 53.9 ± 41.2 |
| *CoVariant spike binding antibody (FI)* |  |  |  |
| SARS-CoV-2 ECD / WT Ab | 4431.3 ± 4480.2 | 7628.9 ± 4961.6 | 7693 ± 5832.1 |
| B.1.1.7 ECD / Alpha Ab | 3492.2 ± 3523.2 | 5979.1 ± 4024.4 | 5689.9 ± 3249.6 |
| B.1.351 ECD / Beta Ab | 3719.2 ± 3784.4 | 6203 ± 4248.3 | 6150.2 ± 4073.7 |
| P.1 ECD / Gamma Ab | 2918.2 ± 3083.8 | 5178.3 ± 3592.4 | 5055.6 ± 3142.9 |
| B.1.617.2 ECD / Delta Ab | 4424.1 ± 4569.2 | 7948.8 ± 5444.4 | 7608 ± 4831.5 |
| B.1.617.3 ECD / Ab | 4041.4 ± 4327.2 | 7368 ± 5064.3 | 6892.1 ± 4285.8 |
| B.1.1.529 ECD / Omicron Ab | 4536.5 ± 4643.7 | 8302.3 ± 6051 | 7639.5 ± 5292.4 |
| BA.2.12.1 ECD / Omicron Ab | 5894.1 ± 5349.1 | 9699 ± 6440.7 | 9381.2 ± 5853.8 |
| BA.4 ECD / Omicron Ab | 6458.4 ± 6054.5 | 11259 ± 7530.9 | 10371.3 ± 6906.5 |
| BA.5 ECD / Omicron Ab | 6049.7 ± 5582.9 | 10187 ± 6540.2 | 9650.1 ± 6300.9 |
| *CoVariant nucleocapsid binding antibody (FI)* |  |  |  |
| Nucleocapsid / WT Ab | 4455.2 ± 5127.5 | 6926 ± 7599.7 | 9028.6 ± 7560.9 |
| Nucleocapsid / Omicron Ab | 2198.5 ± 3003.3 | 2750.5 ± 3439.9 | 3618.7 ± 3146.1 |

**Table S4. The surrogate neutralizing or binding antibody responses in Tainan residences with different medical conditions**

|  | Elderly care center (n=50) | Nursing home (n=51) | Dialysis (n=100) | PLWH (n=45) |
| --- | --- | --- | --- | --- |
| *CoVariant spike neutralizing antibody (nAb)* |  |  |  |  |
| SARS-CoV-2 ECD / WT nAb | 84 ± 24.1 | 84.6 ± 23.4 | 89.3 ± 19.1 | 80.2 ± 22.8 |
| B.1.1.7 ECD / Alpha nAb | 73.4 ± 28.1 | 81.8 ± 23.6 | 85.3 ± 19.7 | 76.7 ± 22.2 |
| B.1.351 ECD / Beta nAb | 75.5 ± 30.9 | 80.9 ± 24.5 | 77.7 ± 26.9 | 69.9 ± 24.1 |
| P.1 ECD / Gamma nAb | 72.5 ± 30.1 | 79.7 ± 26.4 | 79.2 ± 22.4 | 67.5 ± 26.6 |
| B.1.617.2 ECD / Delta nAb | 77.6 ± 30 | 86.1 ± 22.3 | 86.9 ± 19.8 | 78.9 ± 21.1 |
| B.1.617.3 ECD / nAb | 73.3 ± 28.4 | 78.2 ± 28.4 | 88.6 ± 15.6 | 76.5 ± 24.5 |
| B.1.1.529 ECD / Omicron nAb | 66 ± 33.2 | 71.3 ± 32 | 48.9 ± 32.9 | 43.3 ± 34.9 |
| BA.2.12.1 ECD / Omicron nAb | 69.2 ± 33.5 | 75.1 ± 29 | 51.9 ± 33.7 | 43.5 ± 37.6 |
| BA.4 ECD / Omicron nAb | 69.5 ± 32.5 | 72.6 ± 29.6 | 51.3 ± 35.4 | 44.8 ± 36.5 |
| BA.5 ECD / Omicron nAb | 68.1 ± 33.4 | 72.7 ± 29.3 | 51.4 ± 34.1 | 42.8 ± 36.9 |
| *CoVariant spike binding antibody (FI)* |  |  |  |  |
| SARS-CoV-2 ECD / WT Ab | 4320.3 ± 2496.2 | 3820.2 ± 2963.1 | 3333 ± 2133.5 | 6156.3 ± 4759.5 |
| B.1.1.7 ECD / Alpha Ab | 4131.4 ± 2557 | 3116.4 ± 2514.7 | 2396 ± 1569.9 | 5329.4 ± 4226.1 |
| B.1.351 ECD / Beta Ab | 4061.7 ± 2444.8 | 3098.7 ± 2424.1 | 2775 ± 1859 | 4968 ± 3818.6 |
| P.1 ECD / Gamma Ab | 3822.6 ± 2461.1 | 2258.5 ± 1832.9 | 2313.7 ± 1657 | 4340.9 ± 3440.6 |
| B.1.617.2 ECD / Delta Ab | 4571.9 ± 2866.1 | 3619.2 ± 2712.5 | 3523.8 ± 2334.4 | 6714.3 ± 5221.9 |
| B.1.617.3 ECD / Ab | 4084.5 ± 2617 | 3165 ± 2394.2 | 3111.8 ± 1991.1 | 5984 ± 4940 |
| B.1.1.529 ECD / Omicron Ab | 4886.9 ± 3202.9 | 5065.7 ± 4254.8 | 3730.7 ± 2365.5 | 5903.2 ± 4976.3 |
| BA.2.12.1 ECD / Omicron Ab | 5521.1 ± 3384.2 | 5743.5 ± 4487.4 | 4816.4 ± 2899.1 | 7701.5 ± 5736.3 |
| BA.4 ECD / Omicron Ab | 5495.2 ± 3223.3 | 5477.5 ± 4180 | 5303.8 ± 3242.3 | 8594.3 ± 6303.3 |
| BA.5 ECD / Omicron Ab | 5610.6 ± 3469.1 | 5752.6 ± 4537.6 | 4719.7 ± 2872 | 7700.2 ± 5789.2 |
| *CoVariant nucleocapsid binding antibody (FI)* |  |  |  |  |
| Nucleocapsid / WT Ab | 3085.7 ± 3186.5 | 3995 ± 4439 | 3353.6 ± 3584.6 | 4282.3 ± 4440.7 |
| Nucleocapsid / Omicron Ab | 3152.1 ± 2670.2 | 2437.6 ± 3272.6 | 1848.7 ± 2226.3 | 2460.3 ± 3301.6 |


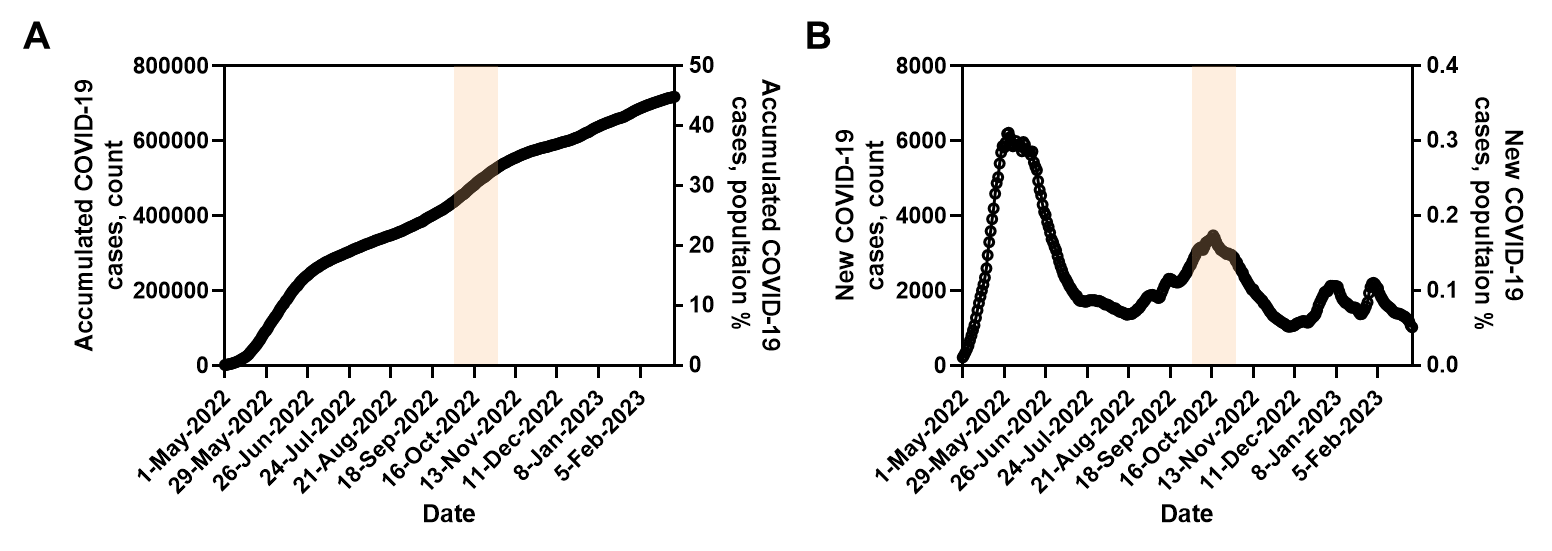


**Figure S1. COVID-19 infection of the whole population in Tainan, Taiwan**

(A) Accumulated COVID-19 cases in Tainan, Taiwan. (B) New COVID-19 cases in Tainan, Taiwan. COVID-19 infection was monitored and reported to CDC in Taiwan from Jan 2020 until March 2023. The yellow box indicates the period of sample collection.


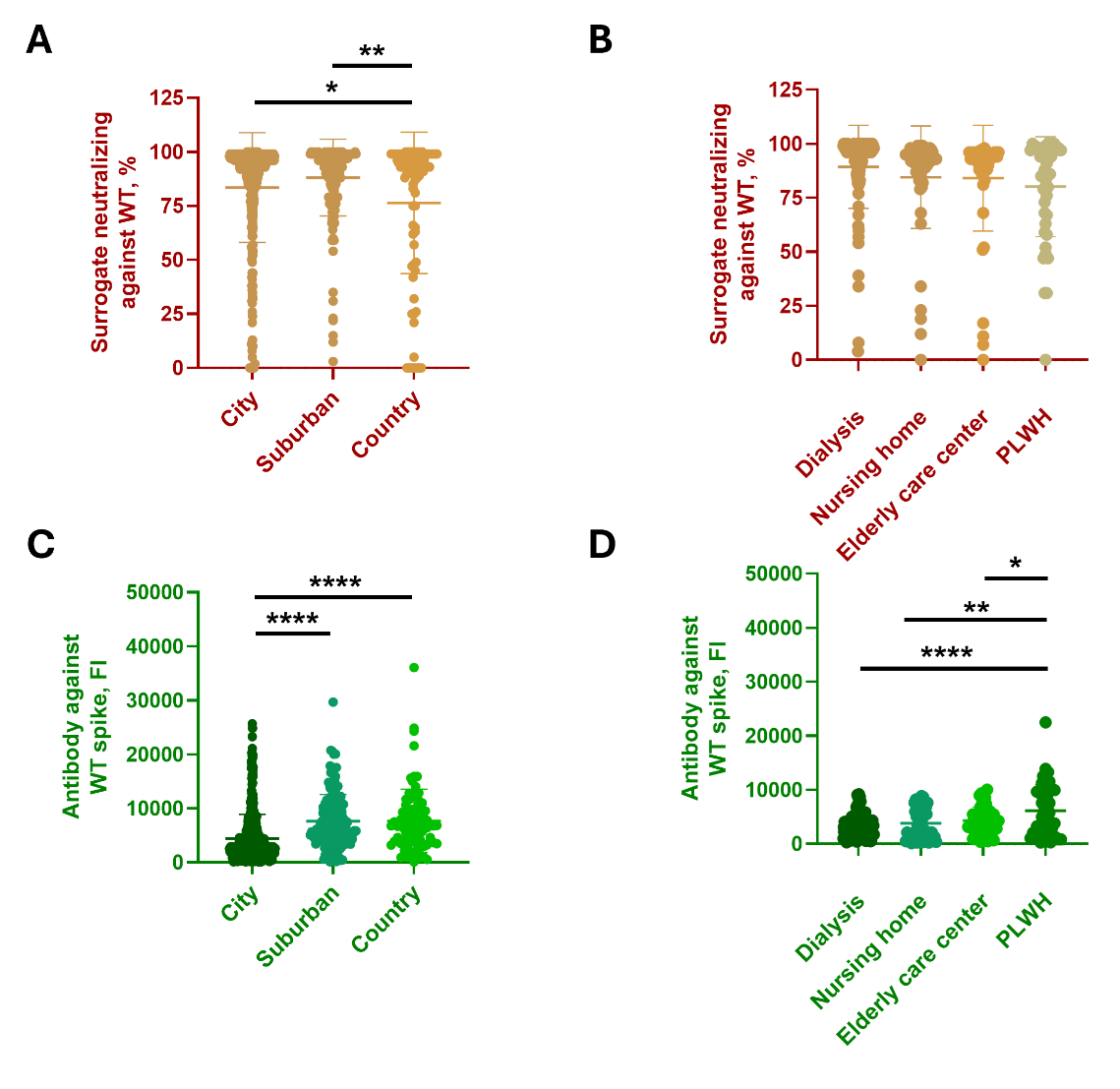


**Figure S2. Neutralizing or antibody against wild-type (WT) ECD in general and special medical care groups.**

Sera from the general population in different Tainan areas and the special medical care groups were analyzed for their neutralizing against WT ECD (A, B) and binding antibody against WT ECD (C, D). Data were analyzed by one-way ANOVA followed by Tukey’s post-tests. *p<0.05 and ****p<0.0001.


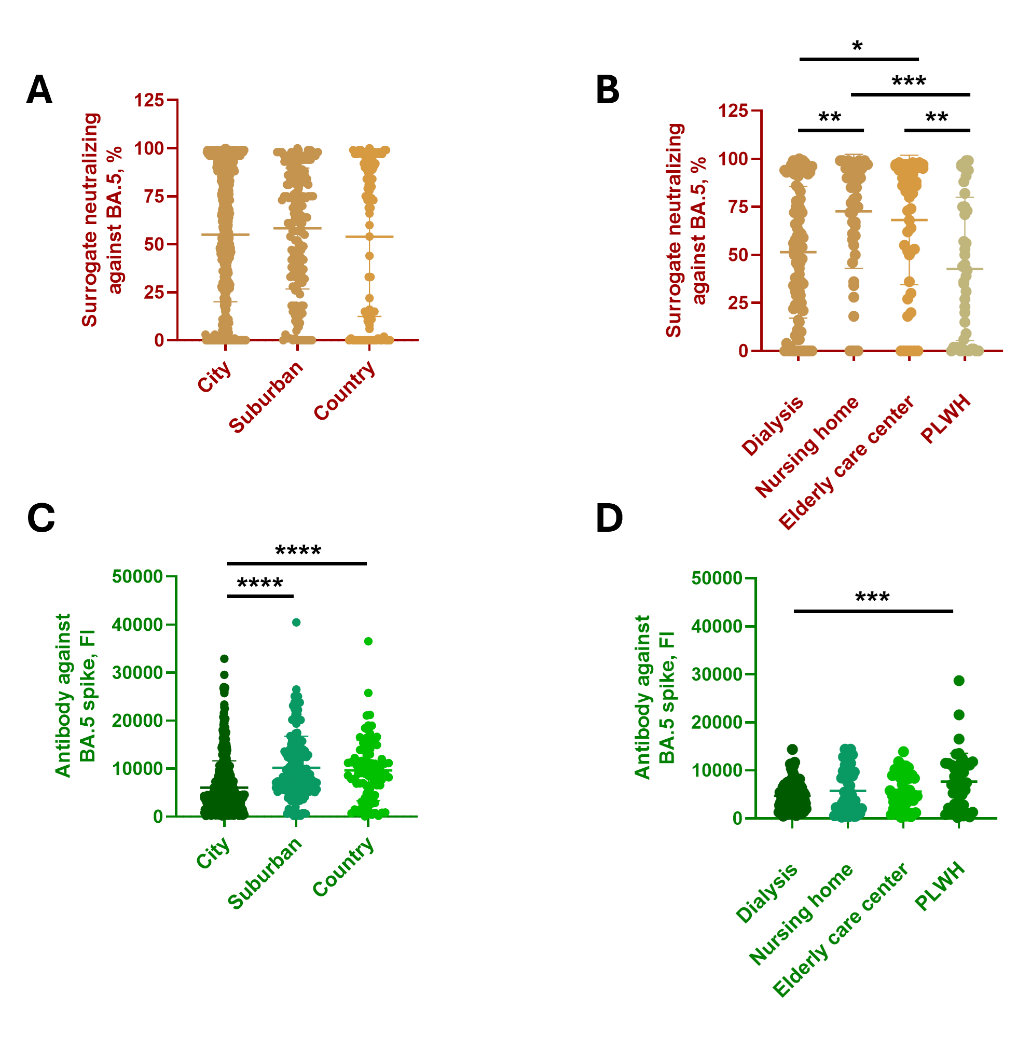


**Figure S3. Neutralizing or antibody against BA.5 ECD in general and special medical care groups.**

Sera from the general population in different Tainan areas and the special medical care groups were analyzed for their neutralizing against BA.5 ECD (A, B) and binding antibody against BA.5 ECD (C, D). Data were analyzed by one-way ANOVA followed by Tukey’s post-tests. *p<0.05 and ****p<0.0001.
